# Functional Induction of the Cystine-Glutamate Exchanger System X_c_^-^ Activity in SH-SY5Y Cells by Unconjugated Bilirubin

**DOI:** 10.1371/journal.pone.0029078

**Published:** 2011-12-27

**Authors:** Pablo J. Giraudi, Cristina Bellarosa, Carlos D. Coda-Zabetta, Paolo Peruzzo, Claudio Tiribelli

**Affiliations:** 1 Centro Studi Fegato, Fondazione Italiana Fegato, Trieste, Italy; 2 Dipartimento Universitario Clinico di Scienze Mediche, Chirurgiche e della Salute, Università Degli Studi di Trieste, Trieste, Italy; Okayama University Graduate School of Medicine, Dentistry and Pharmaceutical Sciences, Japan

## Abstract

We have previously reported that exposure of SH-SY5Y neuroblastoma cells to unconjugated bilirubin (UCB) resulted in a marked up-regulation of the mRNA encoding for the Na^+^ -independent cystine∶glutamate exchanger System X_c_^−^ (*SLC7A11* and *SLC3A2* genes). In this study we demonstrate that SH-SY5Y cells treated with UCB showed a higher cystine uptake due to a significant and specific increase in the activity of System X_c_^−^, without the contribution of the others two cystine transporters (X_AG_^−^ and GGT) reported in neurons. The total intracellular glutathione content was 2 folds higher in the cells exposed to bilirubin as compared to controls, suggesting that the internalized cystine is used for gluthathione synthesis. Interestingly, these cells were significantly less sensitive to an oxidative insult induced by hydrogen peroxide. If System X_c_^−^ is silenced the protection is lost. In conclusion, these results suggest that bilirubin can modulate the gluthathione levels in neuroblastoma cells through the induction of the System X_c_^−^, and this renders the cell less prone to oxidative damage.

## Introduction

Unconjugated bilirubin (UCB) is a bile pigment produced in the catabolism of hemoproteins. Heme oxygenase 1 and 2 catalyzes the rate limiting step in bilirubin formation cleaving heme and obtaining equimolar amounts of Fe^2+^, CO and biliverdin [1], [2]. The ubiquitous biliverdin reductase then reduces biliverdin to bilirubin. UCB is a hydrophobic compound with extremely low water solubility (≤70 nM) [3] and is transported bound to serum albumin to the liver where is conjugated with glucuronic acid in the hepatocytes by the enzyme UGT1A1 and finally secreted into the bile to be eliminated [4].

Newborn infants often show increased plasma levels of UCB. This common condition, known as physiologic jaundice, is generally a benign and transient phenomenon. In some cases, this hyperbilirubinemia can progress to bilirubin encephalopathy ranging from minimal neurological injury to severe and permanent neurodevelopmental dysfunctions, condition knowing as kernicterus [5].

Bilirubin cell toxicity is determined primarily by the concentration of the unbound (free) fraction of UCB (Bf) rather than total bilirubin levels -(unbound and bound to albumin) [6]. Since UCB can diffuse into any cell [7], [8] and it is toxic at high concentrations [9], all cells must maintain the intracellular quantity of UCB below toxic concentrations. Since most cells are unable to conjugate bilirubin, they have to oxidize and/or export UCB to prevent its intracellular accumulation [10].

The precise mechanism of UCB-mediated cellular toxicity remains unknown. Various observations suggest that the damage is initiated at the level of membranes (plasma, mitochondrial, and endoplasmic reticulum (ER)) with resultant perturbations of membrane permeability and function [11]–[13]. These perturbations will contribute to the genesis of neuronal excitotoxicity [14], [15], mithocondrial energy failure [16]–[21] and increased intracellular Ca^2+^ concentration [22]. Collectively, these three phenomena and downstream events trigger cell death by both apoptosis and necrosis. Process like DNA fragmentation, release of cytochrome c, activation of caspase-3 and cleavage of poly(ADP)ribose polymerase has been described in bilirubin cell death by apoptosis [12], [23], [24]. In addition, recent evidences demonstrate that UCB-mediated apoptosis in Hepa 1c1c7 cells is associated with oxidative stress [20] and in HeLa cells, the increase in intracellular reactive oxygen species, due to UCB, activate a signaling pathway involving APE1/Ref-1, Egr-1 and PTEN [25]. The oxidative stress involvement, after overstimulation of glutamate receptors with the consequent increase in nNOS expression and production of *NO radicals, has been recently described [26].

In response to oxidative attack, cells have developed an antioxidant defense system to maintain cellular redox homeostasis and to protect cells from damage. The thiol-containing small molecules (e.g. glutathione), reactive oxygen species-inactivating enzymes (e.g. glutathione peroxidase), and phase 2 detoxifying enzymes (e.g. NAD(P)H: quinine oxidoreductase 1 (NQO1) involved in the reduction of reactive intermediates; γ-glutamate cysteine ligase (GCL) responsible for the biosynthesis of thiols and stress response proteins like heme oxigenase 1 (HO-1)) are members of this antioxidant system [27].

The major cellular antioxidant glutathione is an important line of defense against reactive oxygen species and electrophiles [28]. Glutathione is a tripeptide containing the amino acids cysteine, glutamate and glycine. Its synthesis is limited by availability of the sulfhydryl amino acid cysteine, which is present *in vitro* as cystine in the stock culture media (cysteine half-life: 0.5–1 h in the culture medium) [29], [30]. Both Na^+^-dependent and Na^+^-independent processes are involved in translocation of cystine across the plasma membrane in a wide range of cell types. In the presence of Na^+^, cystine is accumulated into the cells by high-affinity sodium dependent cystine∶glutamate transporter (System X_AG_^−^) whereas, principally in neurons, cystine transport occurs via the sodium independent cystine∶glutamate exchanger (System X_c_^−^) and the sodium independent multifunctional enzyme cystine transporter (GGT) [31], [32].

Structurally, System X_c_^−^ is a member of the disulfide-linked heterodimeric amino acid transporter family and consist of a light-chain subunit (xCT, encoded by the *SLC7A11* gene), which confers substrate specificity [33], and a glycosylated heavy-chain subunit (4F2hc, encoded by the *SLC3A2*) common to the transporter family [34]. It transports cystine into cells in a 1∶1 exchange with glutamate and is inhibited by high concentrations of extracellular glutamate. The activity of System X_c_^−^ is induced by various stimuli, including electrophilic agents like diethylmaleate [35], oxygen [36], bacterial lipopolysaccharide [37], cysteine and others amino acids [38].

Recently, we have reported the effect of UCB exposure of SH-SY5Y neuroblastoma cells on several genes by [39] and shown that 230 genes were induced after 24 h of treatment. Since *SLC7A11* and *SLC3A2* were among the strongest up-regulated genes, we investigated the effect of UCB on both the expression and the activity of the cystine transport systems (X_AG_^−^, X_c_^−^ and GGT). The present data show that UCB elicits a functional activation of the X_c_^−^ transport system which improves the response of the cells to an oxidative stress insult.

## Materials and Methods

### Chemicals

Dulbecco's Phosphate Buffered saline (PBS), streptomycin and penicillin were purchased from Euroclone, Milan (Italy). Ham's Nutrient Mixture F12 (F12), Fetal calf serum (FCS) contained 54 µM albumin and GlutaMAX™ obtained from Invitrogen (Carlsbad, CA). Ham's Nutrient Mixture F12 (F12), Eagle's Minimum Essential Medium (EMEM), nonessential amino acid solution (MEM), dimethyl sulfoxide (DMSO), 3(4,5-dimethiltiazolil-2)-2,5 diphenyl tetrazolium (MTT), L-buthionine-[S,R]-sulfoximine (BSO), NADPH, diethylmaleate (DEM), hydrogen peroxide (H_2_O_2_), glutathione reductase (EC 1.8.1.7), 2,2′-dinitro-5,5′-di-thiobenzoic acid (DTNB), L-quisqualic acid and Tri Reagent were purchased from Sigma Chemical Co.-Aldrich, Milan (Italy).

Chloroform was obtained from Carlo Erba, Milan (Italy) and L-[^14^C]- cystine (9.25 GBq/mmol) was purchased from Perkin-Elmer Life Sciences.

Unconjugated bilirubin (UCB) (Sigma Chemical Co, St. Louis MO) was purified as described by Ostrow & Murkerjee [40]. UCB required to reach the desired Bf (140 nM) was dissolved in 0.6% v/v of DMSO and diluted in complete medium with 15% FCS. Bf was measured as previously described [41].

### Cell culture

SH-SY5Y human neuroblastoma cells (ATCC CRL-2266) were cultured in a mixture of F12-EMEM (1∶1) containing 15% (*v/v*) foetal calf serum, 1% (v/v) non-essential amino acids, 1% (v/v) GlutaMAX™, penicillin (100 U/mL) and streptomycin (100 µg/mL) in 75 cm^2^ tissue culture flasks at 37°C in a humidified atmosphere of 5% CO_2_. The cells were fed every 2 days and subcultured once they reached 80–90% confluence. Cultures were stopped at the 20^th^ passage.

### RNA isolation and quantification by quantitative real time PCR (qPCR)

SH-SY5Y cells were seeded at 6×10^6^ cells in 75 cm^2^ the day before the experiment. Then, cells were exposed to the following experimental conditions: free medium (untreated), 0.6% DMSO medium or 140 nM Bf medium for 1, 4 and 24 h.

Total RNA was isolated by Tri Reagent solution according to the manufacture's suggestions (T9424, Sigma-Aldrich, Milan, Italy). The total RNA concentration and the purity were quantified by spectrophotometric analysis in a Beckman DU640. For each sample the A_260_/A_280_ ratio comprised between 1.8 and 2.0 was considered as good RNA quality criteria. The integrity of RNA was assessed on standard 1% agarose/formaldehyde staining with ethidium bromide gel, indicating that the RNA preparations were of high integrity. Isolated RNA was resuspended in RNAse free water and stored at −80°C until the analysis.

Single stranded cDNA was obtained from 1 µg of purified RNA using the iScrip™cDNA Synthesis Kit, according to the manufacture's suggestions. The reaction was run in a Thermal Cycler (Gene Amp PCR System 2400, Perkin-Elmer, Boston, MA, USA) in agreement with the reaction protocol proposed by the manufacturer.

qPCR was performed according to the iQ SYBER Green Supermix (Bio-Rad) protocol. PCR amplification was carried out in 25 µL reaction volume containing 25 ng of cDNA, 1× iQ SYBR Green Supermix (100 mM KCl; 40 mM Tris-HCl; pH: 8.4; 0.4 mM each dNTP; 40 U/mL iTaq DNA polymerase; 6 mM MgCl_2_; SYBR Green I; 20 mM fluorescein; and stabilizers) (Bio-Rad Laboratories) and 250 nM of gene specific sense and anti-sense primers. qPCR was performed with an iCycler IQ (Bio-Rad Laboratories, Hercules, CA, USA), using β-actin, HPRT and GAPDH as endogenous controls to normalize the expression level of SLC7A11, GGT1 and SLC1A1 genes. Primer sequences and references are reported in *table 1*. The primers were designed using Beacon Designer 4.02 software (PREMIER Biosoft International, Palo Alto, CA, USA).

**Table 1:** List of primer sequences designed for the quantification of specific mRNAs by qPCR.

Gene	Accession Number	Primer Forward	Primer Reverse	Product (bp)
**SLC7A11**	NM_014331.3	GGTGGTGTGTTTGCTGTC	GCTGGTAGAGGAGTGTGC	107
**GGT1**	NM_001032364.1	TCTCTGACGACACCACTC	GACCTTGGAGCCAAAGTAG	152
**SLC1A1**	NM_004170.4	CCACTCTCATTGCTGCTGCTGTTATTC	CATCCACCGTACTGACTTC	120
**β-actin**	NM_001101	CTGGAAAGAATGTCTTGATTGTGG	TTTGGATTATACTGCCTGACCAAG	120
**HPRT**	NM_000194	CTGGAAAGAATGTCTTGATTGTGG	TTTGGATTATACTGCCTGACCAAG	91
**GAPDH**	NM_002046	CCCATGTTCGTCATGGGTGT	TGGTCATGAGTCCTTCCACGATA	145

Cycling parameters were determined and the results were analyzed by using the comparative Ct method as the means of relative quantification, normalized to the housekeeping gene and expressed as 2^−ΔΔCT^. Melting curve analysis and gel electrophoresis were performed to asses product specificity.

### L-[^14^C] Cystine uptake

SH-SY5Y cells were seeded in 6-multiwell plates at a density of 80.000 cell/cm^2^. The day after the cells achieved a 70% confluence and were subjected at 24 h treatment with 140 nM Bf. The cells were then rinsed three times with warmed uptake buffer (140 mM NaCl, 25 mM HEPES, 5.4 mM KCl, 1.8 mM CaCl_2_, 0.8 mM MgSO_4_, 5 mM glucose (pH = 7.5). For Na^+^ -free uptake, NaCl was replaced in the medium with an equal concentration of N-metyl-D-glucamine chloride.

Cystine uptake by SH-SY5Y cells was started by incubating the cells in uptake buffer containing 0.8 µM L-[^14^C] cystine (L- [U-^14^C]-Cystine, 250 mCi/mmol; PerkinElmer Italia Life and Analytical Sciences) at 37°C during 0, 2, 5, 10 and 30 min. For the subsequent experiments the analysis of Na^+^-dependent and Na^+^-independent cystine transport, cells were incubated for a fixed time period of 10 min. The uptake was terminated by rapidly rising cells two times with ice-cold unlabelled uptake buffer. The cells were then lysed by adding 0.8 mL of 0.2 N NaOH containing 1% SDS. An aliquot of 50 µL was taken for protein determination. The remaining solution (750 µL) was mixed with 5 mL of scintillation cocktail (ULTIMA Gold™, Perkin Elmer Italia Life and Analytical Sciences) and the radioactivity was determined using a scintillation counter (Tri-Carb ® Liquid Scintillation Analyzer, Packard Instrument Company).

### Glutathione determinations

SH-SY5Y cells were seeded in 60 mm diameter dish at a density of 80.000 cell/cm^2^. Once the cells achieved a 70% confluence were subjected to the treatment for 24 h with free medium (untreated), 0.6% DMSO medium or 140 nM Bf medium. Then the cells were rinsed three times with PBS at 37°C and 500 µL ice-cold 5% perchloric acid was added. The cells were detached by scrapping, harvested, and the dishes rinsed twice with 500 µL ice-cold 5% perchloric acid. All the fractions were pooled together, homogenized and transferred to eppendorf tubes. The samples were centrifugated at 13.000 g and the acid-soluble fraction was separated from the pellet and both were stored at −80°C until analysis were performed. The proteins were quantified using the bicinchoninic acid assay from acid-precipitated pellet by treatment with 1 M NaOH.

Reduced glutathione (GSH) plus oxidized glutathione (GSSG) were measured as total glutathione content in the acid supernatants using an enzymatic method [42], [43], after its neutralization with a 0.76 M KHCO_3_. Briefly, supernatant aliquots (100 µL) were assessed in 900 µL of the reaction mixture (0.1 M sodium phosphate buffer (pH 7.5) containing 1 mM EDTA, 0.3 mM DTNB, 0.4 mM NADPH). The rate of the enzymatic product formation (Tiobenzoic acid = TNB) was monitored after the addition of glutathione reductase (1 U/mL), in a termostated cuvette (30°C), at 415 nm, for 3 min, with a Beckman DU 640 spectrophotometer. Glutathione concentrations were calculated using appropriate standards and normalized by mg of protein.

### Cell viability of SH-SY5Y Bf treated cells after hydrogen peroxide stress

Cells were plated in 24-multiwell plates. Once they achieved a 70–80% confluence the growing medium was discarded, the cells were rinsed with PBS at 37°C and exposed to medium containing 0.6% DMSO or UCB (Bf 140 nM) for 24 h. Then, the cells were rinsed with PBS and maintained in optimal growth media until hydrogen peroxide treatment was performed. Cell response to hydrogen peroxide (0–700 µM) for 1 h was evaluated immediately after Bf or DMSO treatment and at 156 h upon release in growth medium. After the incubation with H_2_O_2_, the medium was removed and replaced with culture medium containing MTT 0.5 mg/mL for 2 h [44]. Then, medium was discarded and MTT formazan crystals were dissolved by adding 0.4 mL of DMSO. The absorbance values at 570 nm were determined in a LD 400C Luminescence Detector, Beckman Coulter, Milan, Italy. Results were expressed as percentage of MTT reduction respect to cells not exposed to H_2_O_2_.

### Small interference RNA-mediated System X_c_^−^ (SLC7A11) gene silencing

30000 SH-SY5Y cells/cm^2^ (for DMSO treatment) or 60000 SH-SY5Y cells/cm^2^ (for Bf treatment) were plated either in 24-multiwell plates or in 6-multiwell plates for H_2_O_2_ treatment or Western blot analysis, respectively. Cells were untransfected (**MOCK**) or transfected with 50 nM siGENOME Non-Targeting siRNA #1 (Dharmacon, Lafayette, CO, USA) (**NT**, silencing control)n or with 50 nM siGENOME SMARTpool siRNA against human SLC7A11 gene (Dharmacon, Lafayette, CO, USA) (**anti-xCT**). 1.25 µL/well (for 24-multiwell plates) or 5 µL/well (for 6-multiwell plates) of DharmaFECT 1 transfection reagent were used, according to manufacturer's instructions. Media were changed after 24 h, and cells were growth for additional 24 h. At this time, cells were rinsed once with PBS and exposed to medium containing DMSO 0.6% or UCB (Bf 140 nM) for 24 h, as previously described. Cells seeded in 24-multiwell plates exposed to H_2_O_2,_ and viability assessed by MTT-assay. Cells seeded in 6-multiwell plates were lysed on-ice in 200 µL of cell lysis buffer 1× (Cell Signaling, Danvers, MA, USA) supplemented with 1 mM PMSF and the total cell extracts were harvested by scraping. The protein content of xCT in the cell extract was assessed by Western blot analysis using an anti-xCT rabbit polyclonal antibody (ab37185, Abcam, Cambridge, UK) and normalized by actin protein content using an anti-actin antibody (a2066, Sigma, Saint Louis, MO, USA). Bands were analized using Kodak 1D image software and quantified by Scion image software.

### Statistical analysis

All experiments were performed in triplicate and repeated in at least three different cell preparations. Results are expressed as means ± SD. One way ANOVA with Tukey–Kramer post-test was performed using GraphPad InStat version 3.00 for Windows 95 (GraphPad Software, San Diego, CA, USA). Probabilities <0.05 were considered statistically significant.

## Results

### UCB treatment induces the expression of SLC7A11 (but not SLC1A1 and GGT1) in SH-SY5Y cells

**Figure 1:**
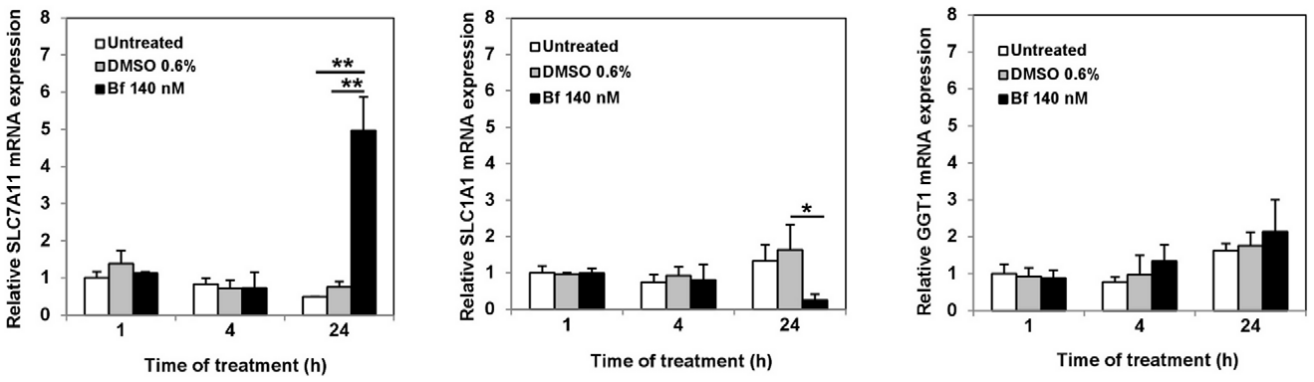
Effect of bilirubin on mRNA expression levels of genes involved in cystine uptake. SH-SY5Y cells exposed to free medium (untreated), 0.6% DMSO medium or 140 nM Bf medium were collected after 1, 4 and 24 h of treatment. Specific mRNA expressions was analysed by qPCR. mRNA expression is shown as mean ± SD of 3 experiments relative to 1 h untreated control set at 1.0. **p<0.001 and *p<0.01 versus DMSO or untreated controls.

SH-SY5Y cells were treated with medium, 0.6% DMSO and 140 nM Bf for 1, 4 and 24 h, and the mRNA expression of the genes involved in cystine uptake (*SLC7A11*, *SLC1A1* and *GGT1*) analyzed by qPCR. As shown in Figure 1, after 24 h of treatment an induction of *SLC7A11* was observed with a mRNA level 5 folds higher (p<0.001) in respect to the controls (untreated cells and 0.6% DMSO); no changes in the expression was observed after 1 or 4 h of UCB treatment. On the contrary, the *SLC1A1* gene expression was decreased (p<0.01) 24 h after UCB treatment while no change respect to controls (0.6% DMSO and untreated) was observed at 1 and 4 h (Figure 1). The expression of GGT1 was not affected by UCB treatment at any time in the study (Figure 1).

mRNA expression of others antioxidant proteins and phase II enzymes, such as gamma-GCS (γ-GCS), heme oxygenase-1 (HO-1) and quinone oxidoreductase 1(NQO1),was also analyzed. The exposure of cells to UCB resulted in an up-regulation of HO-1 (at least 5 folds) only at 24 h and an up-regulation of γ-GCS (at least 4 folds) only at 4 h. No changes on the expression of other enzymes at 1, 4 and 24 h were found (data not shown).

### L-[^14^C] Cystine uptake by SH-SY5Y cells after UCB treatment

**Figure 2:**
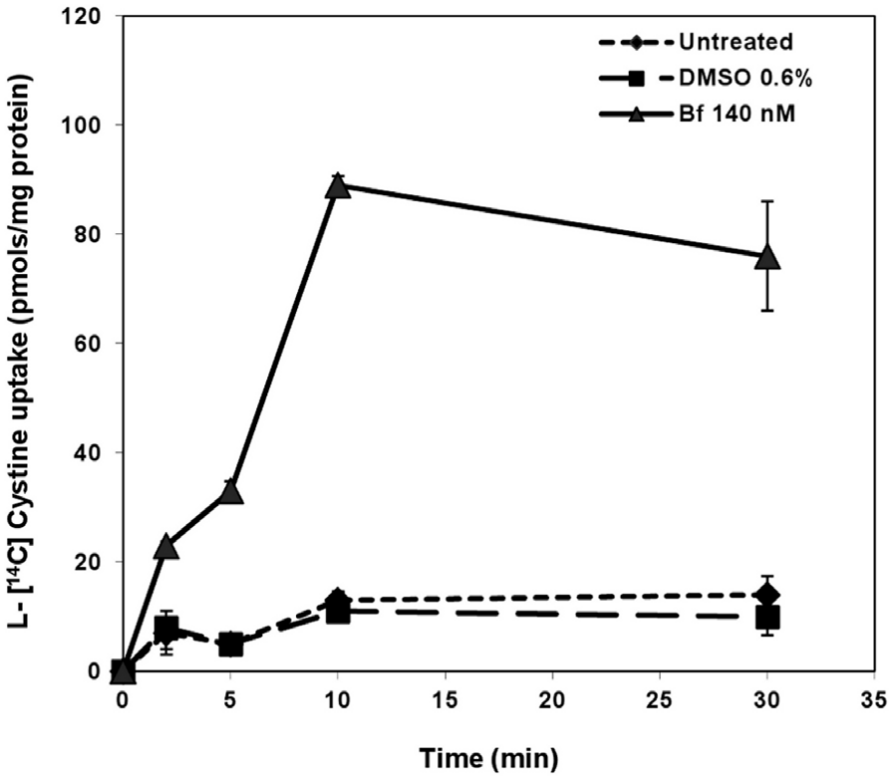
Cellular cystine accumulation in SH-SY5Y cells treated with bilirubin. Cells were exposed for 24 h to free medium (untreated), 0.6% DMSO medium or 140 nM Bf medium for 24 h. At the end of the treatment cells were incubated with L- [ ^14^C] Cystine (0.8 µM) for 0, 2, 5, 10 and 30 min and uptake was evaluated and expressed as pmols/mg protein. Each point represents the mean ± SD (*n = 3)*.

Once shown that SLC7A11 mRNA expression is upregulated by UCB, we examined whether the activity of the System X_c_^−^ was also increased. Untreated SH-SY5Y, exposed to 0.6% DMSO or to 140 nM Bf cells for 24 h were incubated for 0, 2, 5, 10, and 30 min with an uptake buffer containing 0.2 µCi/mL of L-[^14^C] cystine (0.8 µM) and cystine uptake measure therafter. The results are reported in Figure 2. SH-SY5Y cells previously treated with UCB showed a cystine uptake significantly higher than controls (untreated and 0.6% DMSO treated cells) over the course of 30 min. The uptake in Bf treated cells was about 8 times higher than in controls at 10 min and remained elevated thereafter. No difference was observed between controls and cells exposed to DMSO.

### Sodium -dependent and -independent cystine uptake systems in SH-SY5Y cells treated with UCB

Considering that System X_AG_^−^ is a sodium-dependent transporter and System X_c_^−^ and GGT are sodium-independent transporters we examined the relative contribution of each transporter to the overall transport of L-cystine. We also evaluated the contribution of System X_c_^−^ activity in total cystine uptake by determining L-cystine transport by treating the cells with L-quisqualate, a specific inhibitor of System X_c_^−^ [45]. Uptake of L-[^14^C] cystine was measured at 10 min in cells previously exposed for 24 h to UCB (Bf 140 nM); cells exposed to 0.6% DMSO or untreated were used as controls. The experiment was performed in presence or absence of sodium ions (140 mM) and with or without L-quisqualate (500 µM).

**Figure 3:**
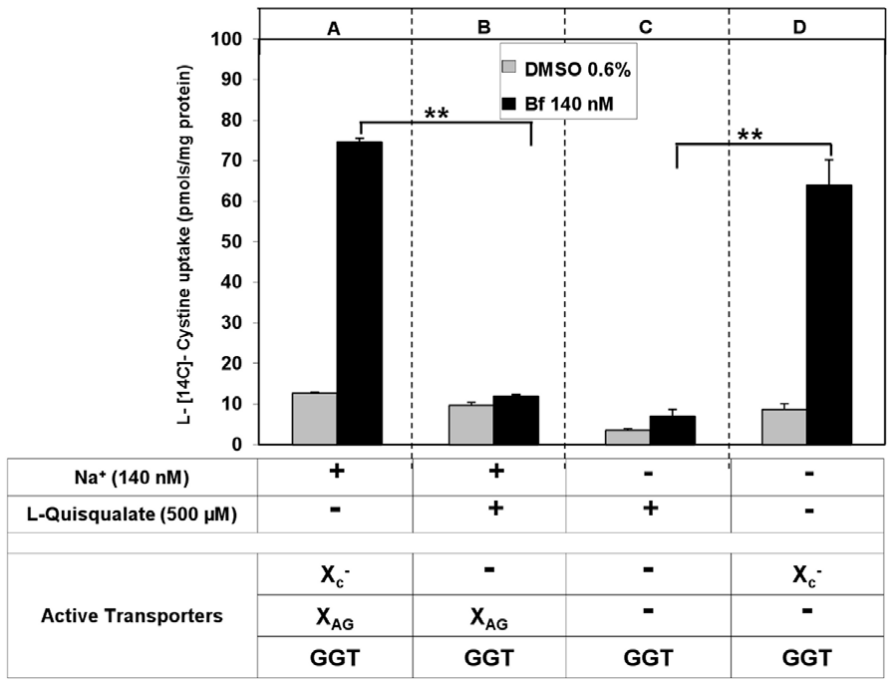
Sodium –dependent and independent L-[^14^C] cystine transport in SH-SY5Y cells. Transport activity was measured in control (0.6% DMSO, 24 h) and treated (Bf 140 nM, 24 h) cells. L-[^14^C] cystine (0.8 µM) uptake was measured after 10 min incubation at 37°C in the presence and absence of sodium ions. L-quisqualate (500 µM) was used as a specific inhibitor for System X_c_^−^. Each point represents the mean ± SD of three plates of cells (** p<0.001).

Figure 3 - panel A, shows that when all the transporters are active (presence Na^+^ and absence of L-quisqualate), cystine uptake was about 8 times higher in Bf treated cells than controls, as also shown in Figure 2. In presence of sodium and after L-quisqualate addition (Figure 3 - panel B), L-cystine uptake in UCB treated cells showed values similar to control cells (from 74.7±0.9 to 11.9±0.2 pmols/mg protein respectively, p<0.001). This result suggests that System X_c_^−^ plays a major role in accounting for the increased cystine uptake induced by UCB treatment.

When sodium was removed from the medium (Figure 3, panel C) but in presence of L-quisqualate (System X_c_^−^ blocked), the sodium dependent System X_AG_^−^ was further inhibited leaving cystine transport accounted only by GGT activity. Under these conditions cystine uptake was not significantly decreased both in controls (from 9.7±0.8 to 3.6±0.5 pmols/mg protein) and treated cells (from 11.9±0.4 to 7.0±1.6 pmols/mg protein). These results indicate that the contribution of System X_AG_^−^ is not modified by UCB treatment. When the activity of System X_c_^−^ is added to that of GGT (absence of both Na^+^ and L-quisqualate), L-cystine uptake was again significantly higher in UCB treated than in control cells (64.0±12.6 pmols/mg protein vs 7.0±1.6 pmols/mg protein, respectively, p<0.001) (Figure 3, panel D), indicating that only System X_c_^−^ activity and not System X_AG_^−^ and GGT activity is induced by bilirubin treatment.

### Effects of UCB treatment on intracellular GSH levels

Total glutathione was determined 1, 4 and 24 h after exposure to140 nM Bf, DMSO and medium, the same time intervals used in the study of the mRNA expression of the cystine transporters (see Figure 1). GSH content was also evaluated after 24 h treatment of the cells with 100 µM BSO or 100 µM DEM. The glutathione extracted from the cells was mostly GSH, and the content of GSSG was negligibly low in these studies, representing only around of 2% of the total gluthathione content [46].

**Figure 4:**
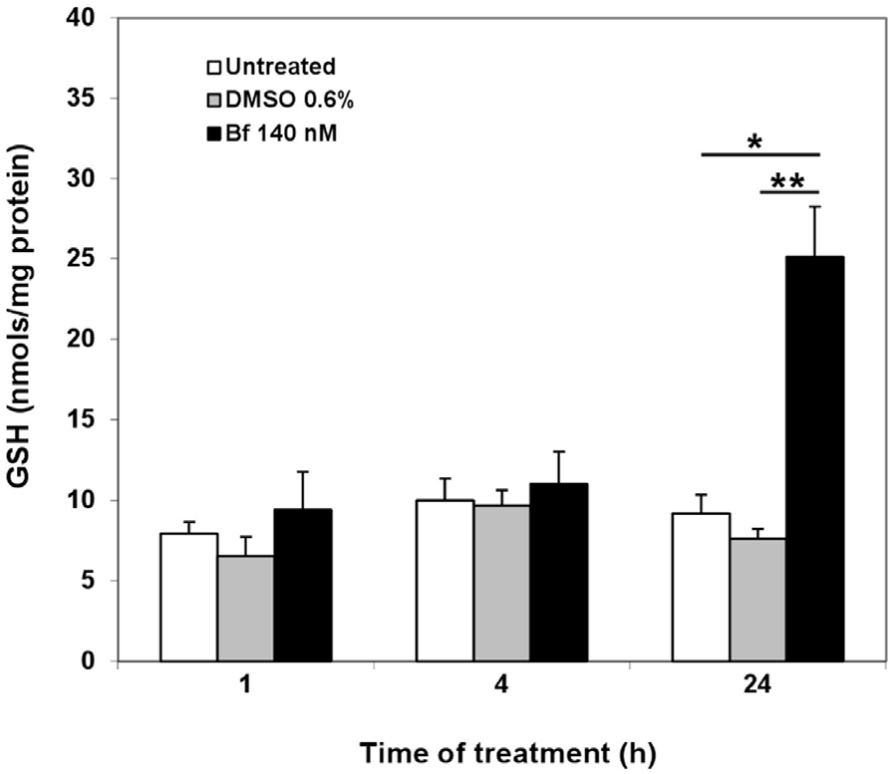
Glutathione levels in SH-SY5Y cells treated with bilirubin. SH-SY5Y cells were exposed to free medium (untreated) or 0.6% DMSO medium or 140 nM Bf medium and total intracellular content of gluthathione was determined after 1, 4 and 24 h. Results are expressed as mean ± SD of 3 independent experiments performed in triplicate. *p<0.01 versus untreated and **p<0.001 versus 0.6% DMSO.

As shown in Figure 4, 1 and 4 h after UCB exposure no changes in GSH levels were observed in SH-SY5Y cells treated with UCB as compared to controls (0.6% DMSO or untreated cells). On the contrary, after 24 h the total GSH content was 2 folds higher in UCB treated cells than controls (25.1±5.3 vs. 7.6±0.6 and vs. 9.2±1.1 nmols/mg protein for DMSO and untreated cells, p<0.01). As expected, GSH content was increased 4 folds after treatment with DEM (GSH = 41.5±5.8 nmols/mg protein) and reduced 10 folds by BSO (GSH = 0.80±0.10 nmols/mg protein). No changes in cell viability after BSO or DEM treatment were detected by MTT test (data not shown) by any of the treatments.

### Response of SH-SY5Y UCB treated cells to oxidative stress

Experiments were carried out to examine if the reductive intracellular environment due to GSH increase content in UCB-treated cells may be protective from an oxidative stress induced by H_2_O_2_ treatment.

**Figure 5:**
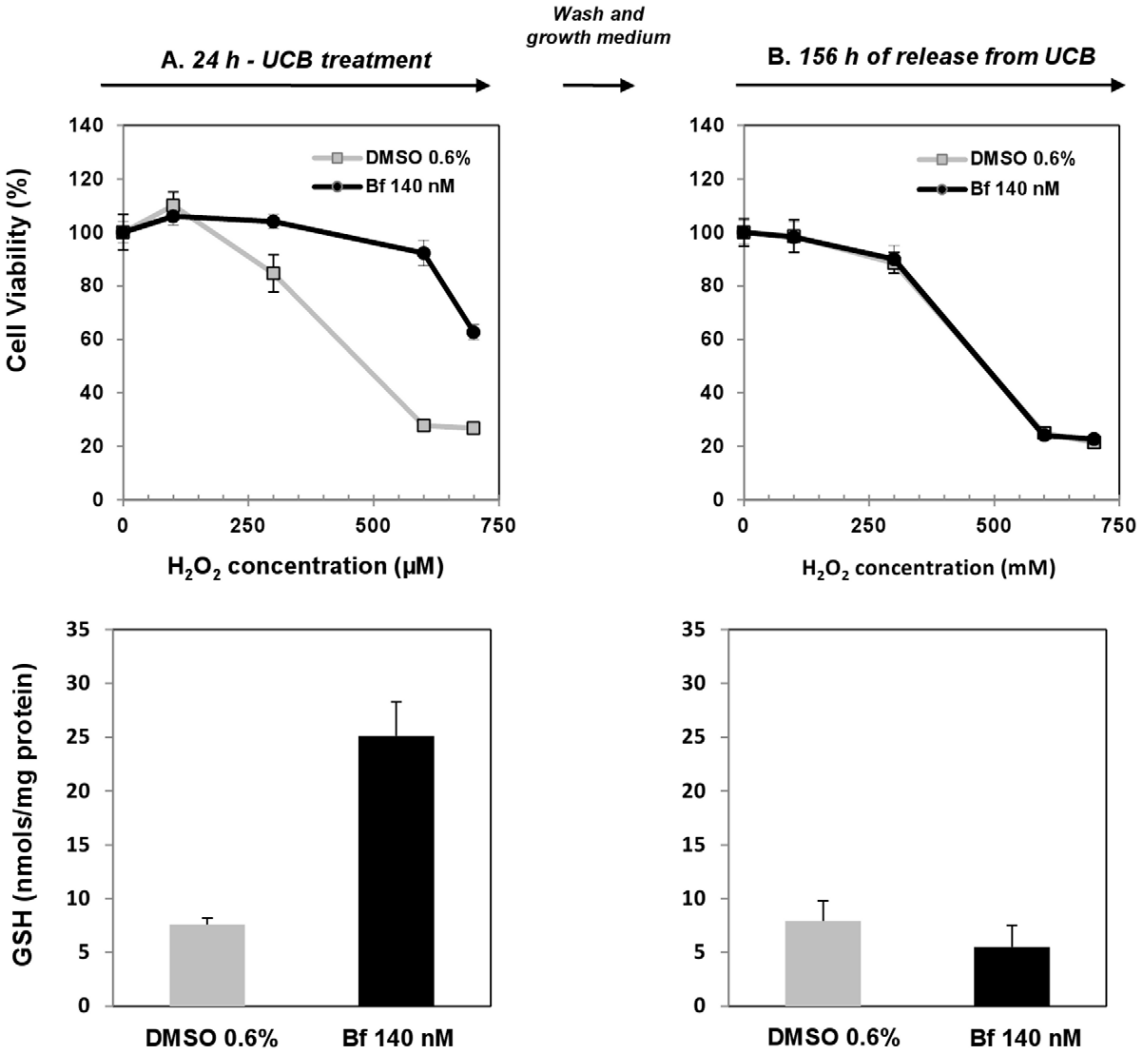
Response to H_2_O_2_ oxidative stress of SH-SY5Y cells pretreated with bilirubin and GSH intracellular levels of the cells before H_2_O_2_ treatment. SH-SY5Y cells were exposed to two successive treatments: the first with 140 nM Bf (control with 0.6% DMSO medium) for 24 h. After this treatment, cells were released in growth medium for 156 h. Immediately after exposure to UCB (column A) and 156 h (column B) of release, cells were incubated, as second treatment, with different concentrations of H_2_O_2_ for 60 min and viability evaluated by MTT. At each time viability is expressed as a percentage of the controls considered as 100%. At each time viability results are compared with GSH intracellular levels of the cells before H_2_O_2_ treatment. Results are expressed as media ± SD of four different experiments.

As shown in Figure 5 (upper panel A), the exposure for 60 min to increasing concentrations of H_2_O_2_, of SH-SY5Y cells previously treated with 0.6% DMSO for 24 h was followed by a dose-dependent reduction in cell viability. On the contrary, cells exposed for 24 h to 140 nM Bf showed a significantly lower damage. At 600 µM of H_2_O_2_ the cell viability of control cells was reduced by 80% while those exposed to UCB were not affected by the treatment. This results are in agreement with the GSH cellular content (lower panel A). Similar responses to H_2_O_2_ have been observed when neuroblastoma cells were pre-exposed for 24 h with 100 µM of DEM, a well known System X_c_^−^ activity inducer (data not shown).

When cells pre-treated with 140 nM Bf for 24 h were left grown for 156 h in the absence of UCB, the sensitivity to the H_2_O_2_-induced oxidative stress was comparable to control cells (upper panel B), and this was associated with GSH cellular level similar in the two cell populations (lower panel B).

### Response of UCB pre-treated SH-SY5Y cells to oxidative stress after SLC7A11 gene silencing

**Figure 6:**
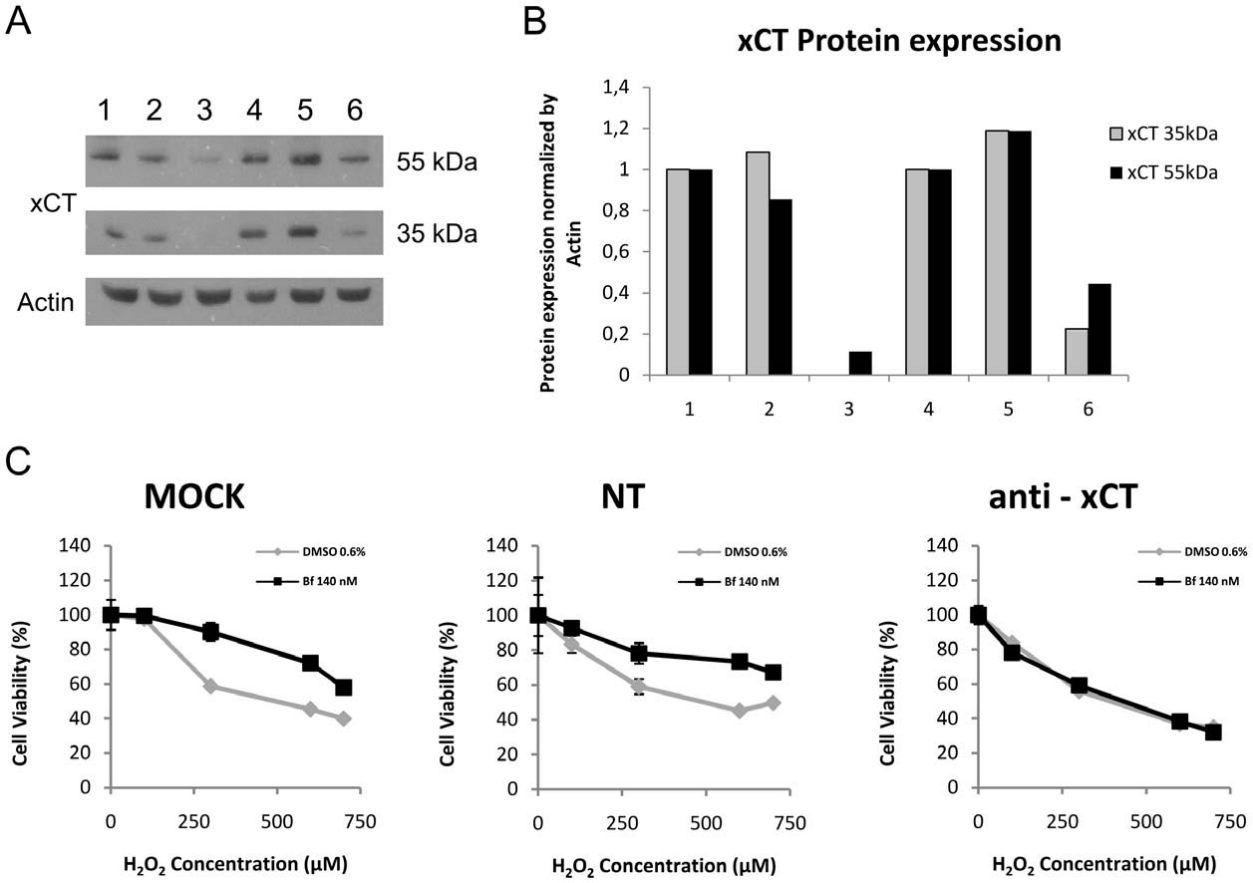
Response to H_2_O_2_ oxidative stress in xCT silenced SH-SY5Y cells pre-treated with bilirubin. Panel A: Protein expression of xCT (55 kDa and 35 kDa) was analyzed by Western blot after siRNA and Bf treatment. Lane 1: MOCK and 0.6% DMSO; lane 2: NT and 0.6% DMSO; lane 3: anti-xCT and DMSO 0.6%. Lane 4: MOCK and Bf 140 nM; lane 5: NT and Bf 140 nM ; lane 6: anti-xCT and Bf 140 nM. Panel B: Quantification of bands shown in lanes from 1 to 6 of panel A. xCT 35 kDa and 55 kDa bands normalized by actin and expressed as relative to MOCK . Panel C) H_2_O_2_ dose-response by MTT test was evaluated in untransfected (MOCK – left), transfected with not targeting siRNA (NT – middle) and transfected with siRNA against SLC7A11 gene (anti-xCT - right) SH-SY5Y cells. After silencing, and before 1 h H_2_O_2_ treatment, SH-SY5Y cells were exposed for 24 h to 0.6% DMSO or Bf 140 nM as indicated in the legend of each picture.

To test the contribution of System X_c_^−^ to the cytoprotective effects induced by UCB we performed experiments gene silencing of SLC7A11 using siRNA. The expression of xCT protein (55 kDa and 35 kDa) was studied in SH-SY5Y cells untransfected (MOCK), transfected with not targeting siRNA (NT) and transfected with siRNA against SLC7A11 gene (anti-xCT) after 24 h of Bf 140 nM or 0.6% DMSO treatment. Two bands were observed, one at 35 kDa and the other at 55 kDa, the former corresponding to the monomeric form of xCT and the latter 55 kDa, even if specific only to xCT if unknown function [47]. As shown in Figure 6A, xCT protein expression was decreased only in siRNA anti-xCT treatments (lanes 3 and 6). The expression of the xCT band of 35 kDa was undetectable in DMSO treated cells (Figure 6B, lane 3) and reduced by 88% in Bf treated cells (Figure 6B, lane 6). Reduction of 55 kDa band was 89% in DMSO treated cells (Figure 6B, lane 3) and 66% in Bf treated cells (Figure 6B, lane 6).

Figure 6C shows the cytotoxic effects of 60 min exposure to increasing concentration of H_2_O_2_ in DMSO or Bf pre-treated cells, after SLC7A11 gene silencing. A dose-dependent reduction in cell viability was observed for MOCK and NT groups. Pre-exposure to Bf resulted in a significantly lower cytotoxicity than DMSO pre-treated cells (p<0.05 at 300 µM H_2_O_2_ and p<0.01 at 600 and 700 µM of H_2_O_2_. On the contrary, in anti-xCT group (Figure 6C), the sensitivity to the oxidative stress was identical between Bf pre-treated cells and controls, supporting a direct contribution of the System X_c_^−^ to the protection against H_2_O_2_ oxidative stress.

## Discussion

In the present study we demonstrate, for the first time, that UCB at a concentration found in jaundiced newborns is an effective inducer of the activity of the System X_c_^−^ in SH-SY5Y neuroblastoma cells. This increase in cystine uptake lead to higher intracellular glutathione levels which protect the cell from an oxidative stress.

We have previously reported an induction of SLC7A11 (xCT subunit) and SLC3A2 (4f2hc subunit) gene expression in SH-SY5Y cells after exposure to 140 nM Bf [39]. Here we extend this study by showing that the up-regulation in the gene expression is associated with a functional activation of the cystine transport system described in literature in both astrocytes and neurons [31], [32], [48]. The cystine transport is accounted by 3 different mechanisms as System X_c_^−^ (SLC7A11), System X_AG_^−^ (SLC1A1) and the multifunctional ectoenzyme GGT (GGT1). Exposure to UCB results in a marked gene and functional up-regulation of System X_c_^−^ while the other 2 systems appear to be unchanged both in terms of expression and activity.

In 1984, Bannai S. [35] described that electrophilic compounds (diethyl maleate, sulfobromophthalein, ethacrynate) at relatively low concentrations, caused an increase in cellular glutathione due to the enhanced uptake of cystine *via* Na^+^ independent transport (designated as System X_c_^−^ by Makowske and Christensen in human fibroblasts) [49]. It is noteworthy that cysteine is readily converted to cystine by autooxidation [50]. Once inside the cell cystine is rapidly reduced to cysteine, which is then incorporated into proteins and glutathione. Because cysteine is a rate-limiting precursor for glutathione synthesis, the intracellular regulation of the glutathione level by the System X_c_^−^ activity is well defined [51]–[53].

Sasaki et. al. [54] demonstrated in BHK21 cells that the activity of System X_c_^−^ was significantly induced by various electrophilic agents like diethyl maleate, arsenite, CdCl_2_, hydroquinone and that the induction of xCT gene (the specific subunit of the System X_c_^−^) was mediated by Keap1/Nrf2 pathway.

Data have been provided indicating the activation through Keap1/Nrf2 pathway of the System X_c_^−^ by several electrophiles together with others genes encoding phase II enzymes (Heme oxygenase-1 or HO-1, NAD(P)H:quinine oxidoreductase or NQO1, Multidrug resistance-associate protein 1 or MRP1, γ-glutamylcysteine synthetase or γ-GCS, glutathione S-transferase or GST, catalase or Cat) [55]–[58].

In our previous transcriptomic analysis [39], we speculate that induction of System X_c_^−^ induced by UCB may represent a major pro-survival response. In the present study we demonstrated that the gene overexpression is functionally effective as associated with a higher activity of the transporter. In a proteomic analysis performed under the same experimental conditions used in this study, Deganuto et. al. [59] described an increase of the expression of proteins involved in cell proliferation (APMAP, APRT, WARS) and that bilirubin decreased cellular growth rate, as a consequence of DNA oxidative lesions. We observed after UCB exposure SH-SY5Y cells start to growth in optimal growth media only 96 h after of release (data not shown). Previous reports by Ollinger R et. al. demonstrated that UCB acts as a natural inhibitor of proliferation in vascular smooth muscle cells and HRT-18 colon cancer cells [60], [61].

The GSH level was significantly higher in treated UCB cells respect to controls immediately after treatment when the cells were not growing. Accordingly, UCB treated cells were able to respond better than control cells to an oxidative stress induced by H_2_O_2_ treatment. GSH content returns to basal levels 156 h after the end of treatment when the cells restart growing and the different oxidative stress response between UCB treated and untreated cells was lost. GSH is recognized as a general regulatory molecule with several functions like the modification of proteins as part of normal cell physiology and signaling [62]–[65]. Recent studies have shown that intracellular reductive environment is the key requirement for allowing DNA synthesis to occur [66].

We showed that cells exposed to UCB and containing high GSH levels were resistant to an oxidative stress induced by H_2_O_2_ treatment. Previous evidence showed that bilirubin is a pigment with antioxidant capacity at low concentration as described by Doré S. et. al. [67] in 1999 by studying the effect of nM concentrations of UCB in the cytoprotection of primary hippocampal cultures to H_2_O_2._ Furthermore, Baranano et al. [68] propone, a biosynthetic cycle wherein oxidize bilirubin is generated from biliverdin by biliverdin reductace in a redox cycle. Recently Sedlak et. al. [69] demonstrated that bilirubin largely protects against lipid peroxidation while GSH primarily prevents the oxidation of water soluble proteins, and the possibility of overlapping between the two mentionated systems.

Our data support the conclusion that UCB exposure may be associated with a protection from an oxidative stress by increasing the GSH cellular content and by modulating stress response protein (i.e., HO-1). Regarding GSH homeostasis we observed an early up-regulation of the rate limiting enzyme for GSH synthesis γ-GCS and an induction of the expression and the activity of the System X_c_^−^ at 24 h. We also demonstrated that the cellular response to and oxidative injury is abolished when xCT is silenced. Increased HO-1 expression occurs under a wide range of unrelated conditions that are characterized by alteration of the cellular redox state [70].

Based on these data, we hypothesize that a subpopulation of SH-SY5Y cell culture may bypass the first cytotoxic damage of bilirubin by a combination of multiple mechanisms which include the System X_c_^−^, maintaining a balanced redox environment through GSH modulation. The surviving neuroblastoma cells not only will restart the cell growth but also will be better suited to respond to an external oxidative stress. Further investigations will be necessary to understand the molecular pathways through which bilirubin activates the System X_c_^−^ in our model.
